# Risk of classic Kaposi sarcoma with exposures to plants and soils in Sicily

**DOI:** 10.1186/1750-9378-5-23

**Published:** 2010-12-02

**Authors:** James J Goedert, Giuseppe Calamusa, Carmelo Dazzi, Anna Perna, Colleen Pelser, Lesley A Anderson, Camille Madsen, Liliana R Preiss, Matt Airola, Barry I Graubard, Angelo Messina, Carmela Lauria, Nino Romano

**Affiliations:** 1 Division of Cancer Epidemiology & Genetics, National Cancer Institute, National Institutes of Health, Rockville, Maryland, USA; 2 Dipartimento di Igiene e Microbiologia 'Giuseppe D'Alessandro', Università degli Studi di Palermo, Palermo, Italy; 3 Dipartimento di Agronomia Ambientale e Territoriale (DAAT), Facoltà di Agraria, Universitá degli Studi di Palermo, Palermo, Italy; 4 Department of Epidemiology and Preventive Medicine, University of Maryland School of Medicine, Baltimore, Maryland, USA; 5 Centre for Public Health, Queen's University Belfast, Belfast, Northern Ireland; 6 Department of Health Science, Brigham Young University, USA; 7 RTI International, Rockville, Maryland, USA; 8 Westat, Rockville, Maryland, USA; 9 Dipartimento di Scienze Biomediche, Università degli Studi di Catania, Catania, Italy; 10 Lega Italiana per la Lotta Contro i Tumori-Sez Ragusa, Ragusa, Italy

## Abstract

**Background:**

Ecologic and in vitro studies suggest that exposures to plants or soil may influence risk of Kaposi sarcoma (KS).

**Methods:**

In a population-based study of Sicily, we analyzed data on contact with 20 plants and residential exposure to 17 soils reported by 122 classic KS cases and 840 sex- and age-matched controls. With 88 KS-associated herpesvirus (KSHV) seropositive controls as the referent group, novel correlates of KS risk were sought, along with factors distinguishing seronegatives, in multinomial logistic regression models that included matching variables and known KS cofactors - smoking, cortisone use, and diabetes history. All plants were summed for cumulative exposure. Factor and cluster analyses were used to obtain scores and groups, respectively. Individual plants and soils in three levels of exposure with *P*_trend _≤ 0.15 were retained in a backward elimination regression model.

**Results:**

Adjusted for known cofactors, KS was not related to cumulative exposures to 20 plants [per quartile adjusted odds ratio (OR_adj_) 0.96, 95% confidence interval (CI) 0.73 - 1.25, *P*_trend _= 0.87], nor was it related to any factor scores or cluster of plants (*P *= 0.11 to 0.81). In the elimination regression model, KS risk was associated with five plants (*P*_trend _= 0.02 to 0.10) and with residential exposure to six soils (*P*_trend _= 0.01 to 0.13), including three soils (eutric regosol, chromic/pellic vertisol) used to cultivate durum wheat. None of the KS-associated plants and only one soil was also associated with KSHV serostatus. Diabetes was associated with KSHV seronegativity (OR_adj _4.69, 95% CI 1.97 - 11.17), but the plant and soil associations had little effect on previous findings that KS risk was elevated for diabetics (OR_adj _7.47, 95% CI 3.04 - 18.35) and lower for current and former smokers (OR_adj _0.26 and 0.47, respectively, *P*_trend _= 0.05).

**Conclusions:**

KS risk was associated with exposure to a few plants and soils, but these may merely be due to chance. Study of the effects of durum wheat, which was previously associated with cKS, may be warranted.

## Background

Kaposi sarcoma-associated herpesvirus (KSHV, also known as human herpesvirus 8) is considered a necessary but insufficient cause of Kaposi sarcoma (KS)[1]. Without overt immunosuppression such as AIDS or allogeneic transplant, the annual incidence rate of classic KS (cKS) after age 50 is only about 6.2/100,000 and 2.5/100,000 for KSHV-seropositive men and women, respectively [2]. Non-smoking, diabetes, and use of corticosteroid medications have 2- to 4-fold effects on the risk of cKS [3,4], but additional cofactors remain to be identified.

Because it has unusual clinical and geographic features, at least four categories of environmental cofactors for KS have been proposed. Noting similarities to podoconiosis, Ziegler postulated that KS may result from volcanic soil chronically embedded in the skin [5]. Mbulaiteye suggested that KS may result from enhancement of T-helper type 2 immunity due to chronic schistosome or other parasite infections [6]. Coluzzi thought that KS may result from alterations of cellular immunity induced by biting flies [7]. Lastly, Whitby postulated that KS may result from increased KSHV lytic replication induced by contact with phorbol esters or other constituents of plants [8].

We conducted a population-based study of cKS in Sicily, where KSHV seroprevalence is approximately 10% [4]. In addition to non-smoking, diabetes, and use of corticosteroid medications, cKS risk was independently increased 2.7-fold with residential exposure to chromic luvisol [9]. Soils are only one component of a complex ecology that includes insects, microbial organisms, and plants. Herein, we began to dissect these issues by investigating whether cKS or KSHV serostatus among controls was related to residential exposure to various soils or to direct contact with plants that have postulated biologic effects.

## Results

The analysis was restricted to 962 subjects: 122 cases, 752 KSHV seronegative controls, and 88 KSHV seropositive controls with childhood residence in a Sicilian community and with complete data on contact with all 20 plants. From the parent study of 1374 subjects, the 412 excluded subjects included 48 with childhood residence outside Sicily, 299 with incomplete plant data, 3 with incomplete cortisone data, 59 controls with indeterminate KSHV serostatus, and 3 with residence in a community that lacked soil data. Table 1 presents the core model with the distributions for sex and age group (the matching variables) and three cofactors for the 962 included subjects. The associations of cKS with non-smoking (*P*_trend _= 0.05), cortisone use and diabetes were similar to those reported previously [4]. Cumulative work with plants or soils (none, ≤900 weeks, >900 weeks) was not associated with cKS (*P*_trend _= 0.81) and thus not retained in the core model.

**Table 1:** Population-weighted multinomial logistic regression model for association of classic Kaposi sarcoma (KS) and KS-associated herpesvirus with core variables.*

		cKS cases		KSHV-negative controls			KSHV + controls*	
***Core model variables***		***N***	OR*	**95% CI**	***N***	OR*	**95% CI**	***N***

Sex								
	Female	45	0.40	0.14 - 1.13	194	0.52	0.20 - 1.37	26
	Male	77	Ref		558	Ref		62
Age								
	81+	29	0.48	0.19 - 1.16	141	0.66	0.30 - 1.45	22
	75 to 80	39	0.67	0.27 - 1.67	174	0.60	0.27 - 1.31	24
	68 to 74	28	0.47	0.19 - 1.16	184	0.54	0.25 - 1.19	24
	<68	26	Ref		253	Ref		18
Smoker								
	Current	9	0.26	0.08 - 0.88	141	0.64	0.25 - 1.66	16
	Former	52	0.47	0.17 - 1.28	329	0.57	0.22 - 1.48	41
	Never	61	Ref		282	Ref		31
Cortisone use								
	Yes	62	1.44	0.75 - 2.77	292	0.94	0.53 - 1.67	38
	No	60	Ref		460	Ref		50
Diabetes								
	Yes	37	7.47	3.04 - 18.35	131	4.69	1.97 - 11.17	8
	No	85	Ref		621	Ref		80

* Odds ratios (OR) and confidence intervals (CI), with KSHV+ controls as referent group, are adjusted for all variables in the model.

### Plant and soil associations with cKS

Adjusted for the "core model" variables, Table 2 presents the risk estimates for cKS in three models that differ in plant categorization and quantification. In the first model, cKS risk was unrelated to cumulative exposure to all 20 plants [per quartile adjusted odds ratio (OR_adj_) 0.96, *P*_trend _= 0.87]. In the second model, cKS risk also was unrelated to uncommon types of plant exposures, as represented in cluster B (OR_adj _2.10, 95% CI 0.83-5.29) and cluster C (OR_adj _0.72, 95% CI 0.19-2.80), compared to the common cluster A. Likewise, in the third model, cKS risk was unrelated to four factors of the plant exposure data, descriptively labeled Asteraceae factor (OR_adj _1.12), Euphorbia/Datura/Agave factor (OR_adj _0.77), Hypericum factor (OR_adj _0.92), and food/beverage/gladiolus factor (OR_adj _1.23, range of *P *= 0.44-0.81).

**Table 2:** Three multinomial logistic regression models for association of classic Kaposi sarcoma (cKS) and KS-associated herpesvirus with aggregate exposures to plants, adjusted for core-model variables.*

	cKS cases				KSHV-negative controls				KSHV + controls*
***Variables***	***N***	OR*	**95% CI**	***P***	***N***	OR*	**95% CI**	***P***	***N***

***Cumulative plant exposure model***									
Lifetime contacts with 20 plants¶									
> 1262	19	0.93	0.35 - 2.44		201	1.73	0.78 - 3.83		16
281 - 1262	34	1.16	0.47 - 2.85	0.87†	189	1.16	0.54 - 2.50	0.04†	24
69 - 280	40	0.97	0.40 - 2.32		183	0.58	0.27 - 1.22		28
0 - 68	29	Ref			179	Ref			20
***Plant cluster model***									
Cluster C‡	4	0.72	0.19 - 2.80	0.64	41	1.17	0.42 - 3.28	0.76	6
Cluster B	21	2.1	0.83 - 5.29	0.11	153	3.01	1.31 - 6.92	0.01	10
Cluster A	97	Ref			558	Ref			72
***Plant factor model***									
Asteraceae factor									
> median	56	1.12	0.58 - 2.19	0.73	390	1.19	0.68 - 2.07	0.55	43
≤ median	66	Ref			362	Ref			45
Euphorbia/Datura/Agave factor									
> median	59	0.77	0.40 - 1.50	0.44	378	0.66	0.37 - 1.17	0.15	49
≤ median	63	Ref			374	Ref			39
Hypericum factor									
> median	65	0.92	0.46 - 1.84	0.81	366	0.87	0.47 - 1.58	0.64	42
≤ median	57	Ref			386	Ref			46
Food/beverage/gladiolus factor									
> median	61	1.23	0.58 - 2.60	0.58	395	1.51	0.79 - 2.89	0.21	40
≤ median	61	Ref			357	Ref			48

* Odds ratios (OR) and confidence intervals (CI), with KSHV+ controls as referent group, are adjusted for the variables in each model, as well as those in the "core model" (Table 1).

¶ The 20 plants were: Agave americana (Agavaceae), Acanthus mollis (Acanthaceae), Ceratonia siliqua (Fabaceae), Chrozophora tinctoria (Euphorbiaceae), Cichorium intybus (Asteraceae), Datura stramonium (Solanaceae), Dittrichia (formerly Inula) viscosa (Asteraceae), Euphorbia characias (Euphorbiaceae), Euphorbia dendroides (Euphorbiaceae), Euphorbia rigida (Euphorbiaceae), Gladiolus communis o italicus (Iridaceae), Hieracium (Asteraceae), Hypericum hircinum (Clusiaceae), Hypericum perforatum guttiferae (Clusiaceae), Iris sisyrinchium (Iridaceae), Lupinus albus (Fabaceae), Matricaria chamomilla compositae (Asteraceae), Picris echioides (Asteraceae), Taraxacum officinale (Asteraceae), and Trigonella foenum-graecum (Fabraceae).

† *P*_trend _values.

‡ Cluster C is numerous plant contacts including Hypericum and Euphorbia. Cluster B is numerous plant contacts other than Hypericum and Euphorbia. Cluster A is relatively few plant contacts.

Table 3 presents the five individual plants and six soils that were associated with cKS risk in the elimination regression model. No other plants or soils met the criterion of *P*_trend _≤ 0.15, adjusted for the core-model and other variables. Results (not presented) differed negligibly when the model was modified by deleting diabetes or by adding asthma history or attained education level. Three plants were associated with elevated risk. One of these, *Taraxacum officinale *(dandelion), had a higher odds ratio (OR_adj _3.59) with <100 contacts than with ≥100 contacts (OR_adj _1.50). The second, *Datura stramonium *(jimson weed), had a high odds ratio (OR_adj _4.26, 95% CI 1.09-16.70) based on only 11 exposed cases. The third, *Lupinus albus *(white lupine), had a high odds ratio with ≥100 contacts (OR_adj _3.58) but marginal significance (*P*_trend _= 0.07). Risk of cKS was significantly lower with *Matricaria chamomilla compositae *(chamomile, *P*_trend _= 0.02), and it tended to be lower with *Acanthus mollis *(bear's breech, *P*_trend _= 0.10).

**Table 3:** Multinomial logistic regression model for association of classic Kaposi sarcoma (cKS) and KS-associated herpesvirus with exposures to individual plants and soils, adjusted for core-model variables.*

	cKS cases				KSHV-negative controls				KSHV + controls*
***Plant and soil variables†***	***N***	OR*	**95% CI**	***P***_**trend**_	***N***	OR*	**95% CI**	***P***_**trend**_	***N***

Taraxacum officinale									
≥100 contacts	9	1.50	0.43 - 5.23	0.04	80	1.71	0.61 - 4.80	0.06	7
<100 contacts	39	3.59	1.60 - 8.03		217	2.03	1.01 - 4.10		16
Zero contacts	74	Ref			455	Ref			65
Datura stramonium									
Any	11	4.26	1.09 - 16.70	0.05	70	2.21	0.63 - 7.79	0.22	5
None	111	Ref			682	Ref			83
Lupinus albus									
≥100 contacts	16	3.58	1.01 - 12.65	0.07	91	2.85	1.00 - 8.08	0.19	8
<100 contacts	72	1.27	0.52 - 3.07		440	1.26	0.63 - 2.53		55
Zero contacts	34	Ref			221	Ref			25
Acanthus mollis									
≥100 contacts	3	0.45	0.08 - 2.53	0.10	39	0.90	0.29 - 2.82	0.92	6
<100 contacts	11	0.53	0.20 - 1.42		119	1.09	0.49 - 2.42		11
Zero contacts	108	Ref			594	Ref			71
Matricaria chamomilla compositae									
≥100 contacts	11	0.29	0.10 - 0.85	0.02	125	0.99	0.44 - 2.22	0.81	12
<100 contacts	64	0.62	0.29 - 1.32		410	0.88	0.47 - 1.64		49
Zero contacts	47	Ref			217	Ref			27
Eutric regosol and/or lithosol									
≥17	28	8.32	2.31 - 29.97	0.01	139	2.35	0.98 - 5.64	0.16	15
<17	15	0.84	0.30 - 2.40		159	1.09	0.49 - 2.43		20
None	79	Ref			454	Ref			53
Chromic and/or pellic vertisol									
≥ 45	33	3.03	0.94 - 9.78	0.04	208	1.33	0.50 - 3.57	0.55	27
< 45	40	2.18	0.72 - 6.61		252	1.92	0.78 - 4.71		32
None	49	Ref			292	Ref			29
Rendzina									
≥15	3	0.16	0.03 - 0.87	0.01	36	0.34	0.10 - 1.13	0.02	5
<15	6	0.41	0.09 - 1.81		43	0.45	0.15 - 1.38		8
None	79	Ref			673	Ref			75
Orthic luvisol									
≥47	35	0.29	0.08 - 1.01	0.01	252	0.63	0.24 - 1.63	0.12	34
<47	36	0.58	0.21 - 1.60		246	1.22	0.28 - 2.81		30
None	51	Ref			254	Ref			24
Vertic cambisol									
≥12	12	0.66	0.15 - 2.79	0.10	127	0.88	0.28 - 2.81	0.18	16
<12	31	0.43	0.15 - 1.22		158	0.23	0.10 - 0.54		29
None	79	Ref			467	Ref			43
Eutric cambisol									
≥157	36	0.22	0.05 - 1.06	0.13	293	0.39	0.12 - 1.31	0.29	43
<157	54	0.79	0.24 - 0.17		300	0.59	0.59 - 1.61		33
None	32	Ref			159	Ref			12

* Odds ratios (OR) and confidence intervals (CI), with KSHV+ controls as referent group, are adjusted for all variables shown, as well as those in the "core model" (Table 1). † Luminescence-weighted soil values in childhood communities, as described in Methods.

Childhood residence in a community with eutric regosol and/or lithosol was associated with an approximately 8-fold higher risk of cKS (*P*_trend _= 0.01, Table 3). Risk also was increased with exposure to chromic and/or pellic vertisol (*P*_trend _= 0.04). Risk of cKS risk was significantly lower with childhood residential exposure to rendzina (*P*_trend _= 0.01) and orthic luvisol (*P*_trend _= 0.01), and non-significantly lower with vertic cambisol (*P*_trend _= 0.10) and eutric cambisol (*P*_trend _= 0.13).

**Figure 1:**
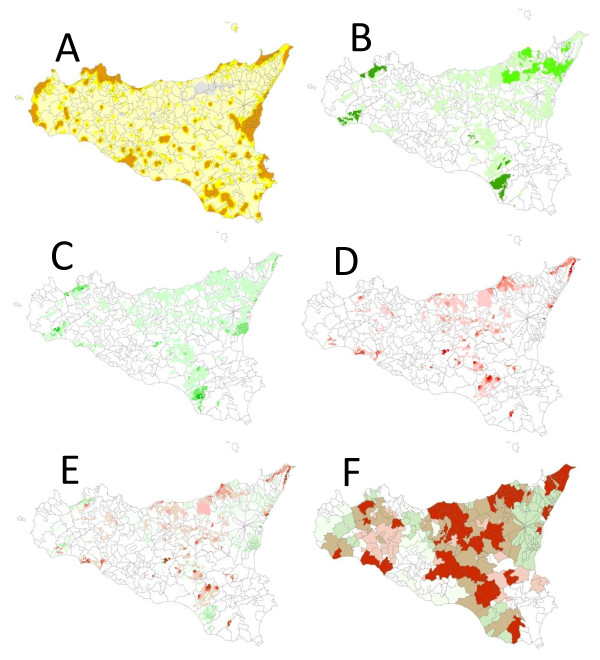
**Political (community boundaries) map of Sicily, with superimposed heat maps of nocturnal luminescence and selected soils**. A. Nocturnal luminescence. B. Orthic luvisol distribution, not weighted for luminescence. C. Luminescence-weighted concentration of orthic luvisol. D. Luminescence-weighted concentration of eutric regosol and/or lithosol. E. Luminescence-weighted concentrations of eutric regosol and/or lithosol (red/pink) and orthic luvisol (green), irrespective of community boundaries. F. Luminescence-weighted concentrations (levels as in Table 3) of eutric regosol and/or lithosol (red/pink), orthic luvisol (green), and overlaps of these (tan) by community

When adulthood, rather than childhood, residential soils were used, the elimination model retained the identical variables shown in Table 3, except eutric cambisol which did not meet the *P*_trend _criterion. Figure 1 illustrates the geography of one high-risk soil (eutric regosol and/or lithosol), one low-risk soil (orthic luvisol), and the overlap of these.

### Associations with KSHV serostatus among controls

As shown in Table 1, history of diabetes was much more common in KSHV seronegative compared to seropositive controls (OR_adj _4.69, 95% CI 1.97 - 11.17). Adjusted for diabetes and the other core model variables, aggregate plant exposure was significantly associated with KSHV seronegativity (Table 2). KSHV seronegatives tended to have more cumulative exposure to the 20 plants (*P*_trend _= 0.04), and they were 3-fold more likely (95% CI 1.31-6.92) to be in cluster B (high exposures to plants other than Hypericum/Euphorbia) than in cluster A (relatively few plant exposures). KSHV seroprevalence was not related to cluster C (high exposures including Hypericum/Euphorbia), nor was it associated with any of the four plant factors (Table 2). When diabetes was eliminated from the model to test for confounding, the associations of KSHV seronegativity with higher cumulative plant exposure and with plant cluster B were essentially unaltered (results not presented). Except for seronegativity with *Taraxacum officinale *(*P*_trend _= 0.06) and seropositivity with rendzina (*P*_trend _= 0.02), none of the individual plants or soils associated with cKS was also associated with KSHV serostatus (Table 3).

## Discussion

Our primary objective was to determine whether exposures to plants or soils were associated with cKS. Neither cumulative nor categorical contacts with plants were related to cKS. Cases with cKS did report more contacts with three individual plants, and they were more likely to have residential exposure to eutric regosol and chromic/pellic vertisol.

For our secondary objective, we found that KSHV seroprevalence among controls was modestly lower with overall exposure to plants (Table 2). This contrasts with our previous observation of higher KSHV seroprevalence with occupational or recreational exposure to plants or soil [10]. Because the earlier seroprevalence analysis was adjusted only for sex and age group, we examined whether the discrepancy might relate to adjusting for diabetes, which was strongly associated with KSHV seronegativity (Table 1). No confounding by diabetes was found. Specifically, exclusion of diabetes from the regression models yielded associations with seronegativity that were almost identical to those presented in Tables 2 and 3.

One soil (rendzina) and one plant (*Taraxacum officinale*) had mirror-image associations with KSHV seroprevalence and cKS risk, which probably appeared by chance. No plant or soil was associated with high seroprevalence and high cKS risk, or with low seroprevalence and low cKS risk.

### Soils and cKS risk

KS risk has repeatedly been associated with soils [5,9,11,12], and an effect of iron has been proposed [13]. Table 4 summarizes the characteristics of the six soils associated with cKS risk in our study [14]. Risk of cKS was elevated in communities with high levels of eutric regosol or chromic/pellic vertisol, all of which are used for cultivation of durum wheat, thereby supporting the higher risk of cKS observed for cereal farmers in Sardinia [15].

**Table 4:** Characteristics of soils associated with risk of classic Kaposi sarcoma (cKS).

	Main features	Main land use	Other Comments
***Soils with higher cKS risk***			
Eutric regosol and/or lithosol	Very shallow soils with an A-C profile (eutric regosol) or shallow soil with an A-R profile (lithosols). Eutric regosol is generally clayey in texture and with a sub-alkaline reaction. Features of lithosols are strongly dependent on those of the parent material on which they evolve.	Natural grazing. Durum wheat is cultivated on Eutric regosol that is not steep.	Prone to erosion. Low agronomic capability.
Chromic and/or pellic vertisol	Deep or very deep soils with an A-Bss-C profile. Texture is clayey throughout and is also characterized by swelling and shrinking processes due to the presence of montmorillonitic clay.	Arable land most commonly cultivated with durum wheat and summertime vegetables (e.g. tomato, watermelon).	Very frequent in flat areas or on gentle slopes. Good agronomic capability, with wide and deep cracks in summertime.
***Soils with lower cKS risk***			
Rendzina	Medium depth soils, A-R and in many cases A-Bw-R in profile, that evolve on limestone or dolomitic limestone substrata. Texture ranges from clay-loamy to loam.	Natural grazing. Improvable by human action for some crops.	Moderate agronomic capability. Uncommon in Sicily.
Orthic luvisol	Moderately deep soils, with an A-Bt-C profile showing a brown argillic B horizon whose color is due to a mixture of clay of different types, organic matter and iron. Texture tends to be clayey in the whole profile, and the amount of clay increases in the Bt horizon.	Vineyards, fruit orchards and, in some cases, vegetables.	Moderate agronomic capability.
Vertic cambisol	Moderately deep soils, with an A-Bss-C profile showing a brown vertic B horizon. Texture is clayey in the whole profile.	Arable land most commonly cultivated with durum wheat.	Moderate to good agronomic capability, with wide cracks in summertime.
Eutric cambisol	Moderately deep soils, with an A-Bw-C profile showing a brown cambic B horizon. Texture is clayey in the whole profile.	Arable land, especially for orchards and vineyards.	Quite good agronomic capability.

Chromic luvisol was associated with cKS in our previous study [9] but not in the current one. Unlike the previous study, the current one simultaneously considered many plants and soils. Of these, orthic luvisol was strongly associated with decreased cKS risk. Areas with luvisols are widely used for vineyards, orchards and citrus groves. Despite this commonality, chromic luvisol generally has a higher content of iron and kaolinite compared to orthic luvisol [14].

Although not offering a simple ecologic pattern, these soil associations can serve to focus future studies. For example, Does cKS risk differ by direct contact with eutric regosol versus orthic luvisol? To address this, much better exposure assessment would be needed. We had only residential data and not occupational or other types of soil exposures. In addition, we collected merely community of residence, not exact location. We used objective data on population density (Figure 1A) to improve the assessment of exposure to soils, but lifetime residential history with exact addresses would be highly desirable.

### Plants and cKS risk

Contact with jimson weed (*Datura stramonium*) was associated with a 4-fold higher risk of cKS; it was not significantly associated with KSHV seroprevalence, but the data were sparse. Higher cKS risk with dandelion (*Taraxacum officinale*) contact was nominally significant but inconsistent with respect to dose-response, and dandelion had a marginal association with KSHV seroprevalence that suggests confounding. The 3-fold lower risk with chamomile (*Matricaria chamomilla compositae*, *P*_trend _= 0.02), which was unrelated to seroprevalence, is noteworthy. Bear's breech (*Acanthus mollis*) had only a marginal association with cKS (*P*_trend _= 0.10). The likelihood of residual confounding by an unmeasured variable, as well as small numbers of exposed cases (for jimson weed and bear's breech) and no difference in cKS risk with cumulative or grouped exposures to plants (Table 2), implies that the 20 plants that we evaluated are irrelevant to the risk of cKS. Notably, we found no associations with any of the four Euphorbia species that we queried, despite the ability of some of their phorbol esters (notably 12-*O*-tetradecanoylphorbol-13-acetate, TPA) to promote tumor growth and induce replication of herpesviruses in vitro [reviewed in refs. [8] and [16]]. We did not ask about contact with durum wheat.

### Strengths and limitations

The strengths of this study include sampling the entire population of the island of Sicily, as well as state-of-the-art KSHV serology and statistical methods. The limitations of our study are several. First, we did not narrowly define an exposure hypothesis. For this reason, exposure was not restricted to dermal contact. Foods and beverages from plants, such as chamomile tea, may have true biologic effects on cKS risk, but they also may be surrogates for socioeconomic or other unmeasured confounding variables. Our latent factor analysis, with one factor heavily weighted to foods and beverages, should have mitigated this problem. Moreover, inclusion or exclusion of education level, as a surrogate for socioeconomic status, did not substantially alter the associations. Second, dermal contact with plants could not be distinguished from dermal contact with soil. Agricultural and gardening work was not related to cKS risk (*P*_trend _= 0.81) [9], but we did not collect data on cereal farming per se [15]. Third, the critical exposure time for a true cKS cofactor is unknown. By including plant exposures occurring over several decades, rather than within a few years of cKS onset, we may have missed a true association. Fourth, although this is the largest cKS case-control study thus far, we had sparse data for some comparisons due to relatively small numbers of cKS cases and KSHV seropositive controls. Finally, some of the associations that we found may have arisen merely by chance from the multiple comparisons that we performed.

## Conclusions

The risk of cKS, compared to KSHV seropositive controls, differed with reported contacts with a few plants and with residential exposure to certain soils. These associations could have arisen by chance due to the multiple comparisons that we performed. Reassuringly, most of these plants and soils were not associated with KSHV serostatus. Future studies might focus on how contacts with farm animals, pesticides and parasites, as well as soils and plants such as durum wheat, affect KSHV viremia, which is strongly associated with risk for KS incidence and progression [17-21]. Associations of cKS and KSHV viremia with human genetic polymorphisms, most of which have not been consistently replicated,[22-25] should also be considered. Understanding these environmental and host interactions will lead to novel insights and means to prevent KS and other herpesvirus-associated malignancies.

## Materials and methods

### Population, Specimen and Data Collection

Detailed methods for the case-control study of cKS in Sicily during 2002-2006 have been published [4]. Briefly, incident cases were ascertained from all histopathology laboratories on the island. Population-based controls, aged 30-99 years were selected using stratified two-stage cluster sampling. As all residents of Italy are assigned to a primary care physician, 450 physicians were randomly selected with the probability proportional to the number of patients on the roster. Up to 12 controls, frequency matched to cKS cases by sex and age in 5-year strata, were selected from each roster.

Institutional review board approval was obtained from the U.S. National Cancer Institute, local institutions in Sicily (Ragusa and Palermo), and the coordinating center (RTI International). Following signed informed consent, recruited participants provided a blood sample and responses to a standardized questionnaire that included demographic, clinical and exposure variables.

### Serologic Classification

KSHV serostatus was defined using immunofluorescence assays (IFA) for antibodies to KSHV lytic and latent nuclear antigens, as well as enzyme-linked immunosorbent assays (ELISA) for antibodies to the KSHV K8.1 and ORF73 gene products [4]. Subjects were considered KSHV seropositive if the latent IFA was positive or the K8.1 optical density (OD) was >1.2. KSHV seronegative was defined as latent IFA negative, K8.1 OD ≤ 0.8, and ORF73 OD ≤ 0.8 [4]. Other controls (n = 59) were seroindeterminate and excluded from the current analysis.

### Classification of Exposure to Plants

Participants were shown color photographs of 20 plants, labeled with common Italian names, and they were asked "Have you ever used or had direct contact with this plant?" Participants who answered "yes" were classified as exposed to that plant. If the participant was uncertain about a particular plant, prompts included common uses of the plant. The 20 specific plant species (listed in footnote of Table 2) were selected on the advice of local botanists based on the prevalence and likelihood of contact in Sicily, known medicinal or cosmetic uses, toxicities, or genetic relatedness to plants reported to induce KSHV lytic replication [8,16]. The questionnaire quantified cumulative exposure, during adulthood, to each plant in categories (zero, <10, 10-100, 100-1000, >1000 contacts).

### Classification of Exposure to Soils

As described previously, exposure to soil was ecologic.(9) Briefly, the questionnaire ascertained each participant's community of residence at birth, during childhood (up to age 12), during adulthood (for 10 years prior to study enrollment), and at enrollment. Exact address was not collected. A map with the boundaries of all 390 communities in Sicily was projected onto the soil map of Sicily [26]. The proportion of each soil was then calculated as the area (the number of pixels) of each soil type in each community. For the current analysis, the previous methods were modified to reduce misclassification of exposure, by weighting for population density in each soil area. Population density was estimated by projecting the map of nocturnal illumination of Sicily (http://ngdc.noaa.gov/dmsp/downloadV4composites.html) onto the soil and community boundary maps (Figure 1A). The type of soil in each pixel (approximately 250 m^2^) was multiplied by that pixel's luminescence (range 0-63, http://www.ngdc.noaa.gov/dmsp/gcv2_readme.txt) (Figure 1C-E), generating luminescence-weighted soil values that were summed for each community (Figure 1F).

### Statistical Analysis Strategy and Methods

The primary objective was to identify cofactors for cKS among people with KSHV infection. The secondary objective was to identify variables that distinguished KSHV seropositive from KSHV seronegative people without cKS. To address these objectives, KSHV seropositive controls were used as the referent group, and the multinomial logistic regression procedure was used to calculate the odds ratio (OR) and 95% confidence interval (CI) for each variable's association with cKS and, among the controls, with KSHV seronegativity.

As described [4], weights were included in each regression model to adjust for the multi-stage sampling of the controls. Base weights were calculated as the product of the reciprocal of the selection probabilities at each stage of sampling. Non-response adjusted weights were then calculated as the product of these base weights and cross-classified categories of age, gender, and (for controls) region (eastern/western Sicily). These non-response adjusted weights were further adjusted by using post-stratification to constrain the weights to reflect the population totals by age, gender and six zones (three community sizes × 2 regions). These non-response/post-stratification-adjusted weights, that were rescaled to sum to the sample sizes of the cases and controls, are the final sample weights for each participant's data. PROC MULTILOG in SUDAAN statistical software (SAS-Callable SUDAAN Release 10.0.1, Research Triangle Institute) was used to conduct weighted multinomial logistic regression analyses that incorporated the sample weights and accounted for the stratified cluster sampling of the controls.

Prior to considering plant and soil exposures, a core model was developed with 5 variables: sex and age category (<68, 68-74, 75-80, ≥81 years) to account for matching variables, plus diabetes, use of oral or topical corticosteroid medication in past 10 years, and cigarette smoking (current, former, never). Cumulative time working with plants or soils, previously noted to be associated with elevated KSHV seroprevalence among women [9,10], was considered but not retained in the core model. All plant and soil analyses were built on this core model, and all models included the identical participants. To assess confounding, plant and soil models were repeated with exclusion of the one core variable (diabetes) found to be associated with KSHV seronegativity. History of asthma [3], level of attained education [4], and both of these were added to the final model to further assess possible confounding or effect modification.

To evaluate how exposures to multiple plants might relate to cKS risk, three dimension-reducing methods were employed. Total contacts with all 20 plants, assuming values of 0, 2, 20, 200, and 2000 for each plant for the exposure categories (zero, <10, 10-100, 100-1000, >1000), were summed (range of values, 0 - 23,224) then divided into quartiles for regression analysis.

Factor analysis uses covariance relationships among multiple observed variables to generate a few underlying, but unobservable, quantities called factors. Four factors were generated with an orthogonal rotation method (VARIMAX and PROC FACTOR, SAS Institute, Cary, NC) based on the proportion of variance explained in the exposures to the 20 plants. These factors were labeled descriptively (Asteraceae, Euphorbia/Datura/Agave, Hypericum, and food/beverage/gladiolus) based on the interpretation of the factors from their factor loadings. The score for each factor was dichotomized at its median value for inclusion as an independent variable in the multinomial regression analysis.

PROC FASTCLUS in SAS was used to partition participants into clusters based on the Euclidean distances computed from the levels of contact with the 20 plants. The uncommon clusters, labeled C (high exposures including Hypericum and Euphorbia) and B (high exposures to plants other than Hypericum and Euphorbia), were compared to the more common cluster (relatively few plant exposures).

For 14 typical soils, the likelihood of each participant's exposure was categorized as none (childhood community with zero for soil or luminescence), low (<median of non-zero luminescence-weighted soil value) or high (≥ non-zero median). For two widely distributed soils (lithosol and eutric regosol) that were present in nearly all communities (<200 controls with zero exposure), tertiles of luminescence-weighted values were used. One uncommon soil (gleyic arenosol) was dichotomized as any versus no exposure.

Lastly, all 20 plants in levels (zero, <100, ≥100 contacts; except any/none for *Datura stramonium*, *Euphorbia characias euphorbiaceae*, *Hypericum perforatum guttiferae*, and *Hypericum hiricinum *to which fewer than 20 participants reported ≥100 contacts) and all 17 soils (classified as in the preceding paragraph) were included in a backward-elimination stepwise regression model. In addition to 5 variables in the core model, individual plants and soil with *P*_trend _≤ 0.15 were retained. As a sensitivity analysis, childhood residential soil exposures were substituted with adulthood soil exposures. Overlaps of the soils that were strongly associated with cKS risk were illustrated (Figure 1E and 1F). In all models, *P *≤ 0.05 was considered statistically significant.

## Abbreviations

AIDS: (Acquired Immunodeficiency Syndrome); CI: (confidence interval); cKS: (classical Kaposi sarcoma); ELISA: (enzyme-linked immunosorbent assays); IFA: (immunofluorescence assays); KS: (Kaposi sarcoma); KSHV: (KS-associated herpesvirus).

## Competing interests

The authors declare that they have no competing interests.

## Authors' contributions

JJG designed the study, obtained funding, supervised the overall project, and drafted the manuscript. GC managed recruitment, coordinated shipments, and collected the questionnaire data and blood specimens. CD provided the soil map data and the utilization of the soils (Table 4). AP processed the blood specimens and performed the KSHV immunofluorescence assays. CP, LAA and CM performed statistical analyses. LRP managed the data and performed the final statistical analyses. MA obtained the luminescence data and constructed the maps. BIG proposed the clustering, factoring, and multinomial logistic regression approaches and supervised the final statistical analyses. AM supervised the processing of specimens and the laboratory activities in eastern Sicily. CL helped to select the plants and supervised field activities in eastern Sicily. NR supervised the laboratory and field activities in western Sicily. All authors contributed to and approved the final manuscript.
